# Massive Facial Presentation of Dermatofibrosarcoma Protuberans

**DOI:** 10.1155/2022/2953579

**Published:** 2022-04-30

**Authors:** Sebastiaan Hermans, Karlijn Clarysse, David Emmanuel Las, Johan de Mey, Maurice Yves Mommaerts, Yannick De Brucker

**Affiliations:** ^1^Department of Radiology and Medical Imaging, University Hospital Brussels, Laarbeeklaan 101, B-1090 Brussels, Belgium; ^2^Vrije Universiteit Brussel (VUB), Universitair Ziekenhuis Brussel (UZ Brussel), Department of Dermatology, SKIN research group, Laarbeeklaan 101, 1090 Brussels, Belgium; ^3^European Face Centre, University Hospital Brussels, Laarbeeklaan 101, B-1090 Brussels, Belgium

## Abstract

Dermatofibrosarcoma protuberans is a low-grade cutaneous sarcoma typically located on the trunk or proximal extremities. Less common locations include the head, face, and neck area. This tumour is slow growing with variable clinical appearance. It is known for its locally invasive nature and low metastatic propensity. Because imaging findings are rather nonspecific, biopsy is needed for definite diagnosis. This case describes an unusually large example of dermatofibrosarcoma protuberans in the less common preauricular region.

## 1. Introduction

Dermatofibrosarcoma protuberans (DFSP) is a rare, locally aggressive sarcoma of the skin that ranges in presentation from a small firm subcutaneous nodule over a coalescent indurated plaque to a giant exophytic nodular mass, depending on the growth duration [1]. The mean size of the lesion at presentation is 4.4–4.9 cm [2]. It mostly occurs between the second and fifth decade of life and is most frequently located in the trunk region or the proximal extremities and less commonly in the head and neck region. If it occurs in the head and neck region, it typically affects the scalp. DFSP grows in an infiltrative manner and has the capacity for local recurrence, though it rarely metastasizes [1]. A rare case of a giant facial DFSP has been described by Kumar et al. [3]. This report details an even larger example of this sarcoma in the preauricular region.

## 2. Case Report

**Figure 1:**
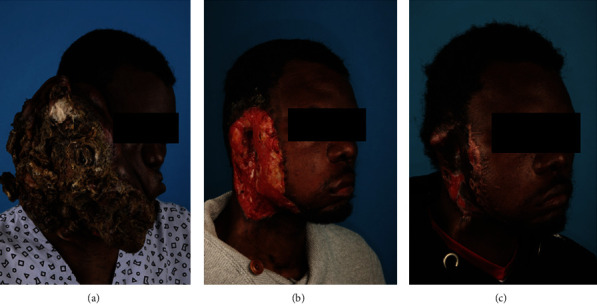
Photographs of a large right-sided facial necrotizing mass causing facial disfigurement after successive treatment. (a) Initial presentation. (b) After reconstruction of first tumour debulking with cutaneous allograft. (c) After reconstruction of the second debulking with split-thickness skin graft.

A 36-year-old African man presented to the emergency department (ED) with a large right-sided facial mass causing massive facial disfigurement. The patient's right ear was displaced superiorly while his mouth was deviated to the left (Figure 1(a)). The foul-smelling tumorous tissue appeared necrotic with purulent and bloody excretions. The mass had been growing over the past eight years, and the patient had undergone multiple resections in Africa, including a right parotidectomy prior presentation at our ED. In addition, the patient had a fever as high as 38.5°C without other systemic symptoms.

Contrast-enhanced computed tomography (CT) of the head and neck showed an exophytic and lobulated mass (dimensions, 28.0 × 9.5 × 23.5 cm) centred at the right preauricular region. It appeared as a moderately hypoattenuated lesion with moderate contrast enhancement without calcifications. In addition, a mass effect was shown on the surrounding tissues with bone scalloping of the right posterior mandibular ramus, the external auditive canal, and the mastoid, all of which are indicative of a slow-growing process. CT angiography showed multiple branches of the external carotid artery circumferentially encased by tumoral tissue. The right upper lobe showed findings compatible with an active open tuberculosis (TB), which most likely was causing the fever in this patient.

**Figure 2:**
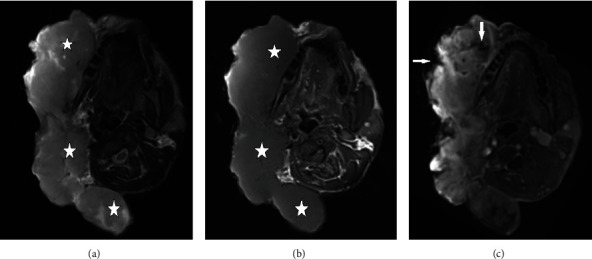
Axial MRI of the head showing a large lobulated mass centered at the right preauricular region. (a) The lesion appearing hypointense on T1-weighted imaging, (b) moderately hyperintense on T2-weighted imaging, and (c) showing marked homogeneous contrast enhancement with peripheral and intratumoral zones of necrosis (white arrows) on T1-weighted imaging after gadolinium administration. The masseter muscle and perioral muscles are not separately visible due to deep muscular invasion. There is also deep invasion of the tumour behind the right mandibular ramus in the masticator space.

On magnetic resonance imaging (MRI), the tumour appeared hypointense on T1-weighted imaging and moderately hyperintense on T2-weighted imaging and showed marked homogeneous contrast enhancement with peripheral zones of necrosis (Figures 2(a)–2(c)). The adjacent right masseter muscle, medial and lateral pterygoid muscles, and the perioral muscles were not separately visible due to deep muscular invasion. In addition, there was anteroinferior displacement of the submandibular gland and the prestyloid and poststyloid parapharyngeal space without clear signs of invasion.

Positron emission tomography with CT showed F-18 fluorodeoxyglucose accumulation in the right facial tumour and in the right upper lobe (due to TB). However, there were no signs of metastasis.

Based on the imaging findings and the location of the tumour, we posed a differential diagnosis consisting of chronic tuberculous lymphadenitis, plexiform neurofibroma, liposarcoma, DFSP, and giant pleomorphic adenoma. Histopathological examination showed an unencapsulated but well-defined nodule consisting of monomorphic spindle cells in a storiform pattern. Immunohistochemistry for CD34 was strongly positive. These findings revealed the diagnosis of DFSP.

**Figure 3:**
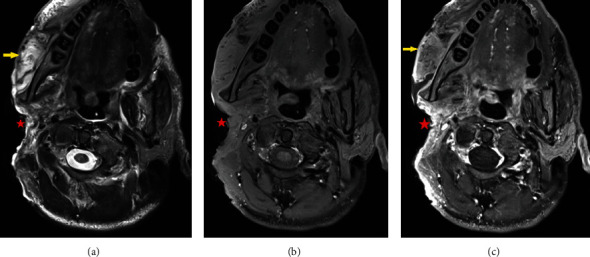
Axial MRI of the head showing postoperative outcome by after the first stage of debulking with a drastic reduction in tumour tissue (red star). (a) T2-weighted imaging, (b) T1-weighted imaging, and (c) T1-weighted imaging after gadolinium injection. The masticator muscles show increased T2w intensity and diffuse enhancement, due to muscle oedema (yellow arrow). Residual tumour tissue is difficult to differentiate for which follow-up imaging is necessary.

Given the tumour's massive size and proximity to important structures, the patient underwent a debulking excisional surgery, a mass of 2.8 kg tumorous tissue was removed in total (Figure 3). The tumour debulking included the excision of the right ear and was restored by means of a transposition flap of the masseter muscle to cover the buccal communication (Figure 1(b)).

A second reconstruction using a split-thickness skin graft from the right thigh was used to cover the remaining defect after two weeks of granulation of the original wound bed (Figure 1(c)). The resected specimen showed diffuse tumoral involvement of the peripheral and deep margins on histopathology. Therefore, curative adjuvant radiation therapy was performed on the resection site after complete healing of the skin graft.

## 3. Discussion

Our case describes a 28 cm diameter DFSP in the head-and neck area of a 36-year-old black male from African origin. Incidence rates have been described as nearly twice as high in black people compared to white people [4]. Its most common locations are the trunk (50–60%) and proximal extremities (20–30%) whereas the head and neck region (10–15%) is far less common [1].

DFSP is a slow-growing tumour that usually starts as an asymptomatic firm subcutaneous nodule or a coalescent indurated cutaneous plaque but can progressively turn into a more lobulated, exophytic mass. DFSP can arise in healthy skin or in areas exposed to repeated trauma, vaccination, radiation, or scarring. At later stages, ulceration and bleeding of the tumorous tissue are possible, and the invasion of adjacent fascia, muscles, and bone has been described. The latter was also observed in our case. Although DFSP is typically locally aggressive, metastasis is rare and occurs in only less than 5% of the cases, typically spreading to the lungs [1, 3].

Imaging findings of DFSP are rather nonspecific. According to Millare et al., CT demonstrates an isoattentuated to hypoattenuated lesion without calcifications and variable contrast enhancement depending on its size. Due to the slow-growing nature of the tumour, the adjacent underlying bone will remodel rather than get aggressively destroyed. For preoperative reasons, radiologists should mention any invasion of the deep head and neck structures in the radiological report. MRI findings include a hypointense signal on T1-weighted imaging and a hyperintense signal on T2-weighted imaging with uniform contrast enhancement on T1-weighted imaging after administration of gadolinium, similar to that seen in our patient [1].

The main differential diagnosis of DFSP consists of dermatofibroma, leiomyosarcoma, Merkel cell carcinoma, keloid, or other soft tissue tumour. Due to the atypical presentation of our case and his positive TB status, our differentials also included chronic tuberculous lymphadenitis, plexiform neurofibroma, liposarcoma, and giant pleomorphic adenoma. A more heterogeneous aspect of the tumour with central cystic changes would be expected in chronic tuberculous lymphadenitis. The absence of the diagnosis of NF-1 in our case makes the diagnosis of plexiform neurofibroma less likely, though not impossible [5]. Lastly, the infiltrative character, as observed in the presented case, is not typical of a giant pleomorphic adenoma [6].

DFSP is a histopathological diagnosis and cannot be made solely on the base of clinical appearance and imaging. Histopathological examination typically shows spindle cells which are irregularly organized in linked fascicles with a storiform arrangement. Furthermore, the spindle tumour cells are positive for CD34 and vimentin on immunohistochemistry [7].

The gold standard treatment of DFSP should aim for complete surgical excision of the lesion by means of wide local excision or MOHS. However, these treatment options were not feasible in our massive and complicated case. First, the head and neck region does not easily allow for wide local excision due to the presence of important surrounding structures such as the carotid artery or base of skull. Second, the enormous size of our patient's tumour warrants debulking rather than curative surgery and therefore largely excludes the option of MOHS. In brief, the challenge in our case's surgical planning was to find a balance between maximal tumour tissue resection and achieving a structural and aesthetical acceptable result despite significant perioperative soft tissue loss. Curative radiation therapy should be offered in the case of unclear surgical margins, as in our case. Adjuvant radiotherapy should also be offered in cases of free surgical margins to prevent early local disease recurrence [3, 8, 9]. It has been described that the local recurrence rate of DFSP is rather high (32–76%) [3].

## 4. Conclusion

DFSP in the head and neck region is characterised by a slow-growing pattern, locally aggressive in nature with high recurrence rates, and low malignant potential. DFSP is usually hyperintense at T2-weighted imaging with marked enhancement at T1-weighted imaging. The current case stands out because of its massive size and unusual off-midline location at the preauricular area. Radiologists should be familiar with the imaging appearance of this entity and include DFSP in the differential diagnosis of head and neck cutaneous tumours with similar characteristics.

## Data Availability

The data used to support the findings of this study are available from the corresponding author upon request.
